# Low Temperatures Affect the Physiological Status and Phytochemical Content of Flat Leaf Kale (*Brassica oleracea* var. *acephala*) Sprouts

**DOI:** 10.3390/foods11030264

**Published:** 2022-01-19

**Authors:** Dunja Šamec, Valentina Ljubej, Ivana Radojčić Redovniković, Stjepana Fistanić, Branka Salopek-Sondi

**Affiliations:** 1 Department of Food Technology, University Center Koprivnica, University North, Trg Dr. Žarka Dolinara 1, 48 000 Koprivnica, Croatia; 2 Department for Molecular Biology, Ruđer Bošković Institute, Bijenička 54, P.O. Box 180, 10 002 Zagreb, Croatia; kruk.valentina@gmail.com (V.L.); stjepana.fistanic@skole.hr (S.F.); salopek@irb.hr (B.S.-S.); 3 Faculty of Food Technology and Biotechnology, University of Zagreb, Pierottijeva 6, 10 000 Zagreb, Croatia; irredovnikovic@pbf.hr

**Keywords:** flat leaf kale, low temperature, phytochemicals, carotenoids, polyphenols, glucosinolates

## Abstract

Consumption of plants in the juvenile stage becomes popular because sprouts are easy to grow, and they can be a tasty source of micro- and macro-nutrients and various phytochemicals. However, some environmental factors during sprout growth can affect their characteristics. In this article, we investigated how low temperatures during cultivation (8 °C) and additional exposure to freezing temperatures (−8 °C) affect the physiological status and phytochemical content of kale (*Brassica oleracea* var. *acephala*) sprouts compared to the control grown at 21 °C. We conducted five independent laboratory experiments and found that low temperature significantly increased proline content and decreased sprouts yield. In addition, low temperature caused a significant decrease in carotenoid and flavonoid content, while phenolic acid content and total glucosinolates content increased, but individual glucosinolates were differentially affected. Our results indicate that low temperatures affect the physiological status of kale sprouts and affect the content of phytochemicals.

## 1. Introduction

The consumption of plants in their juvenile stage, such as sprouts and microgreens, has become popular in recent years due to their richness in nutrients and bioactive compounds that have the potential to prevent malnutrition and chronic diseases [1]. Sprouts are easy to grow and can be an easy and tasty source of micro- and macro-nutrients and various phytochemicals throughout the year [2,3,4]. According to Le et al. [5], vegetables consumed at the juvenile stage are divided into two groups: sprouts and microgreens. Sprouts are obtained from germinated seeds that grow for 2–7 days, constitute shoots and roots, are harvested when the cotyledons are still under developed and the true leaves have not yet emerged, while microgreens may be recognized by the full expansion of the cotyledon leaves and the appearance of the first true leaves that generally occurs within 7–21 days after sowing [5]. During extensive growth and development, plants accumulate phytochemicals faster and several studies have shown that sprouts and microgreens contain more phytochemicals than mature plants/vegetables [6,7].

Sprouts and microgreens of vegetables from the Brassicaceae family, often referred to as cruciferous or mustard family, constitute the most extensive reservoir of genetic resources for commercial production of microgreens and sprouts [8]. Most vegetables from this family belong to the genus *Brassica*, to the species *B. oleracea* (broccoli, cauliflower, cabbage, kale, colard, Brussels sprouts, etc.) or *B. rapa* (pak choi, Chinese cabbage) [9]. They are known by the common name cruciferous and are considered as a good source of dietary fiber, vitamins, minerals and phytochemicals, especially from the group of polyphenols, carotenoids and glucosinolates [2,9,10,11,12]. Research has shown that cruciferous vegetables accumulate even more of these health-related constituents when they are young. For example, Pasko et al. [7] reported that the content of glucosinolate sulforaphane, oleic and linoleic acids is significantly higher in broccoli sprouts compared to florets. In a study investigating the content of polyphenols, flavonoids and glucosinolates in seeds, sprouts and leaves of Tuscan black kale (*B. oleracea* ssp. *acephala* var. *sabellica*), the highest content of phytochemicals was observed in 10-day-old sprouts [12]. Drozdowska et al. [13], who directly compared young shoots and mature plants of red cabbage, reported that 14-day-old ready-to-eat young shoots, which are in the phase of intensive growth, are a better source of proteins, selected minerals and especially glucosinolates than mature cabbage. In another study, direct comparison of sprouts, microgreens and baby leaves of *B. oleracea* showed that sprouts had the highest total phenolic content [14]. According to the in vitro studies, sprouts or microgreens of Brassicaceae may be useful in preventing oxidative stress and inflammation-related diseases [15], cancers [16], liver damage [17], and type 2 diabetes mellitus [18].

The study of *B. oleracea* plants from the *Acephala* group, comparing different kale and collard greens, has attracted worldwide attention and recognition of these vegetables as superfoods in recent years which cause significant increase in their production and consumption [11]. This group of cruciferous vegetables is known for their good environmental adaptation and better resistance to unpleasant conditions such as drought, salt stress, or high and low temperatures [19,20]. Consumption of young kale in the form of sprouts, microgreens, and baby leaves is also becoming increasingly popular and has been reported to be a good source of different phytochemicals [21,22]. In our previous study, kale sprouts showed higher content of polyphenols, glucosinolates and antioxidant activity than broccoli, Chinese cabbage and arugula sprouts [2].

It is well known that different external growth factors such as biotic and abiotic stressors, light, air and substrate composition can affect growth, development, micro-, macronutrients and accumulation of phytochemicals in plants [22,23,24]. Sprouts, as a plant in the juvenile stage, are even more sensitive to changes in growth conditions, so various external factors have been studied to improve growth and phytochemical content. Recently, Almuhayawi et al. [23] reported that laser light enhanced growth, photosynthesis, respiration, and accumulation of primary and specialized metabolites in mustard, cauliflower, and turnip sprouts. According to the review article by Zhang et al. [24], LED light is an efficient and promising strategy for the production of sprouts and microgreens with higher nutritional value. Treatment of sprouts with different molecules elicitors may also lead to a change in the phytochemical content of the sprouts [25,26].

Germination temperature and growing conditions may also affect sprouts yield and nutritional quality [27,28]. Temperature is an important factor that alters yield, quality and phytochemical content in plants. For example, Amfolo et al. [28] reported that a temperature of 30 °C can enhance germination of common beans and increase nutritional value of beans organic edible sprouts. However, as far as we know, low temperature has not been well studied as a factor that can affect the content of phytochemicals in sprouts. Therefore, in the present study, we investigated how low temperature (8 °C) affects yield, pygments and phytochemical (polyphenols, carotenoids, glucosinolates) content of kale sprouts (*B. oleracea* var. *acephala*).

## 2. Materials and Methods

### 2.1. Germination, Growth Conditions and Low Temperature Tretments of Plants

Seeds of kale (*Brassica oleracea* var. *acephala*) were purchased from the Srđan Franić family farm, Vrgorac, Croatia. Seeds were sterilized in 3% Izosan^®^ (Pliva, Zagreb, Croatia) and mixed at 300 rpm for 10 min, washed several times with sterile distilled water and transferred to Petri dishes (20 seeds per dish) containing 1% agar (*w*/*v*) in a laminar flow hood. Plates were first stored in the dark at 4 °C for 72 h and then transferred to a growth chamber where they were maintained at 21 °C for an additional 24 h at a 16/8 (light/dark) h photoperiod in vertical position. Thereafter, only the germinated seeds were transferred to new Petri dishes containing 1% agar.

**Figure 1:**
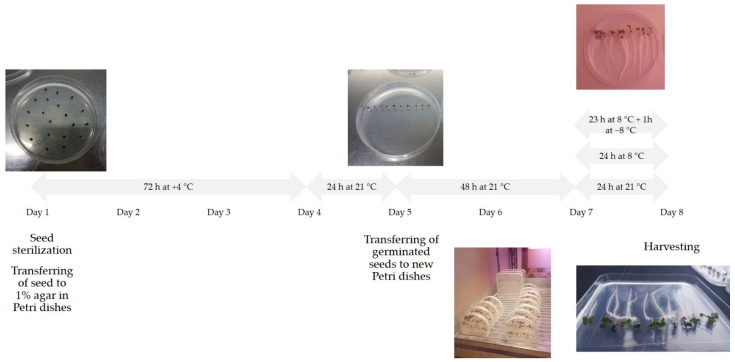
Shematic diagram of the experimental design.

We prepared 30 Petri dishes for each experiment. All plates were kept in vertical position for another 48 h at 16/8 h photoperiod at 21 °C. Then, 10 plates remained at the same temperature (control), and 20 plates were transferred to a chamber at 8 °C under the same photoperiod conditions and vertical position. After 23 h of being at 8 °C, 10 plates were transferred to a chamber at −8 °C under the same photoperiod conditions and vertical position and remained at −8 °C for 1 h. Plant material from 30 plates (control, 8 °C and −8 °C) were harvested at the same time. We performed 5 independent experiments under identical conditions, as 5 biological replicates. The experimental design is shown in Figure 1.

### 2.2. Yield and Length of Sprouts

Immediately after the low temperature experiments and before harvesting, we determined the root and shoot length of all sprouts (using a ruler). We also weighed ten sprouts together and determined the average mass per sprout and expressed yield per sprouts in milligrams (mg). Before measuring, we removed the seed coats if they remained on the sprouts.

### 2.3. Sprouts Storage, Draying and Homogenization

Harvesting was performed by carefully pulling the entire sprouts (including shoots and roots) out of the agar plates with laboratory tweezers. All sprouts were harvested at the same time, wrapped in aluminum foil, and immediately frozen in liquid nitrogen and stored at −80 °C until freeze-dried. All samples were freeze-dried (Lyovac GT2, SRK Systemtechnik GmbH, Riedstadt, Germany) at the same time for 48 h until all water was sublimated. The freeze-dried samples were homogenized into fine powder using liquid nitrogen with mortar and pestle before extraction.

### 2.4. Determination of Proline Content

The proline content in sprouts was determined using 1% ninhydrin [29]. Extraction was performed using 30 mg of the freeze-dried tissue in 70% ethanol. After centrifugation, 100 µL of the supernatant was mixed with 1000 µL of the reaction mixture (1% ninhydrin [*w*/*v*], 60% acetic acid [*v*/*v*], and 20% ethanol [*v*/*v*]) and then heated to 95 °C for 20 min. Proline levels were measured at 520 nm using a UV–VIS spectrophotometer (BioSpec−1601 E, Shimadzu). Proline standard was used for the calibration curve and the results are expressed in μmol g^−1^ dw (micromole per dry weight).

### 2.5. Determination of Pigments

For determination of pigments we used methods reported by Lichtenthaler and Buschmann [30] with small modification. Approximately 10 mg of the freeze-dried plant material were extracted (by vortexing and centrifugation) with 80% acetone until plant material was discolored. Pigments levels were measured at three different wavelengths, 663.2 nm for chlorophyll *a*, 646.8 nm for chlorophyll *b*, and 470 nm for carotenoids, and concentrations were calculated using equations reported by Lichtenthaler and Buschmann [30].The results for chlorophyll *a*, chlorophyll *b*, total chlorophylls were recalculated per dry mass and expressed in mg g^−1^ dw. We also calculated the ratio of chlorophyll *a*/chlorophyll *b* and total chlorophylls/total carotenoids as indicators of physiological processes in plants.

### 2.6. Determination of Polyphenolic Compounds

Extractions of polyphenolic compounds were carried out in a Mixer Mill MM 400 (Retsch, Haan, Germany) for 5 min at 30 Hz using 60 mg of freeze-dried tissue in 2 mL of 80% methanol, followed by 10 min sonication and 1 h mixing at 15 rpm on tube rotator. After centrifugation (10 min 13,000 rpm) supernatant was transferred to a new tube. 

Total phenolic compounds were determined by colorimetric reaction with Folin–Ciocalteu reagent [31]. The added reagent volumes were proportionally reduced so that the final reaction volume amounted to 2 mL and could be prepared in disposable plastic cuvettes. In brief, 20 μL of extract, 100 μL of the Folin–Ciocalteu phenol reagent was added, followed by 300 μL of saturated sodium carbonate solution and made up to the final reaction volume of 2 mL with distilled water. Absorbance was read at 765 nm after 2 h. Gallic acid was used for the calibration curve and the results are expressed as gallic acid equivalents per dry weight (mg GAE g^−1^ dw). 

For the determination of total flavonoids we usedthe methods of Zhinshen et al. [32] adopted to small scale volume. To 200 μL of extract, 800 μL of distilled water and 60 μL of (5%, *w*/*v*) NaNO_2_ were added. Then, 5 min later, 60 μL of (10%, *w*/*v*) AlCl_3_ were added. After additional 6 min, 400 μL of 1 M solution of NaOH were added and the final reaction volume was adjusted to 2 mL with distilled water. Absorbance of the mixture was determined at 510 nm. The Catechin was used for the calibration curve and results are expressed as catechin equivalents per dry weight (mg CE g^−1^ dw). 

Total phenolic acids were determined using Arnow’s reagent [33]. In a plastic cuvette 300 μL of extract was mixed with 300 μL of distillated water, 100 μL of 0.5 M hydrochloric acid and 100 μL Arnow’s reagent (prepared by dissolution of 10 g of sodium nitrite and 11.7 g sodium molybdate dihydrate in 100 mL of water). After mixing of solution in every cuvette 100 μL of 1 M sodium hydroxide and 100 μL of distillated water were added. Absorbance was measured at 505 nm. Caffeic acid served as the standard for generating the calibration curve and results were expressed as caffeic acid equivalents per dry weight (mg CAE g^−1^ dw).

### 2.7. Determination of Glucosinolates

Total glucosinolates were determined spectrophotometrically using a method reported by Aghajanzaden et al. [34]. Approximately 30 mg of freeze-dried tissued were mixed with 80% methanol and heated in a thermobloc at 95 °C for 2 min in order to inactivate myrosinase. After cooling 30 µL of supernatant was mixed with 900 µL of 2 mM disodium tetrachloropalladate (Na_2_PdCl_4_) and absorbantion was measured at 425 nm using UV–VIS spectrophotometer. A sinigrin standard was used for the calibration curve, and results were expressed as milligrams of sinigrin equivalent per gram of dry weight (mg SE g^−1^ dw). 

We also determined individual glucosinolates using HPLC-DAD and ISO methods as reported previously [35]. Freeze-dried tissue (30 mg) was twice extracted with 900 μL of 70% methanol at 70 °C for 15 min. After centrifugation, recovered extracts were passed through an ion-exchange resin Fast DEAE Sepharose CL-6B microcolumn for desulphation with purified sulphatase (from Helix Pomatia) and left overnight at the room temperature. Then, samples were eluted with 1.5 mL of deionized water and analysed on a ZORBAX C18 column (250 mm × 4.6 mm id; particle size 5 μm) using a Perkin–Elmer Series 200 HPLC system (Waltham, MA, USA). Mobile phase consisting of water (A) and 20% acetonitrile in water (B) was used with constant flow rate of 1 mL min^−1^ was employed with gradient elution: 0–1 min 100% A, 1–30 min linear gradient change to 100% B, 30–35 min linear gradient change to 100% A and 35–40 min 100% A. Detection was performed with a UV-Diode Array Detector at 229 nm. Positive identification of desulphoglucosinolates was accomplished by comparing elution order with the retention time of a sinigrin and internal standard glucotropeolin based on ISO standard method for determination of glucosinolates content (ISO, 10633-1:1995) and UV-DAD peak spectral analyses. Individual glucosinolates were recalculated from HPLC peak areas using the response factors to correct the absorbance differences between the internal standard (glucotropeolin) and other identified glucosinolates (ISO, 10633-1:1995). Representative chromatograme is available at Figure S1. The results are expressed as μmol g^−1^ dw.

### 2.8. Statistical Analysis

We conducted five independent experiments, with each experiment as a biological replicate. Results are expressed as mean ± standard deviation (SD). All statistical analyses were performed using the free software PAST [36]. One-way ANOVA and post hoc multiple means comparison (Tukey’s HSD test) were performed and differences between measurements were considered significant at *p* < 0.05.

## 3. Results

### 3.1. Influence of Low Temperature on Sprouts Length and Yield

The mass per sprout and the root and shoot length of the sprouts grown at 21 °C and 8 °C are shown in Table 1. Sprouts exposed to additional temperature at –8 °C did not differ from those at 8 °C because they were exposed to freezing temperature for only 1 h, too short a time to observe differences, so we did not report these data here. As expected, low temperature significantly reduced sprouts mass and root and shoot length by 13%, 35%, and 11%, respectively.

### 3.2. Proline Content

**Figure 2:**
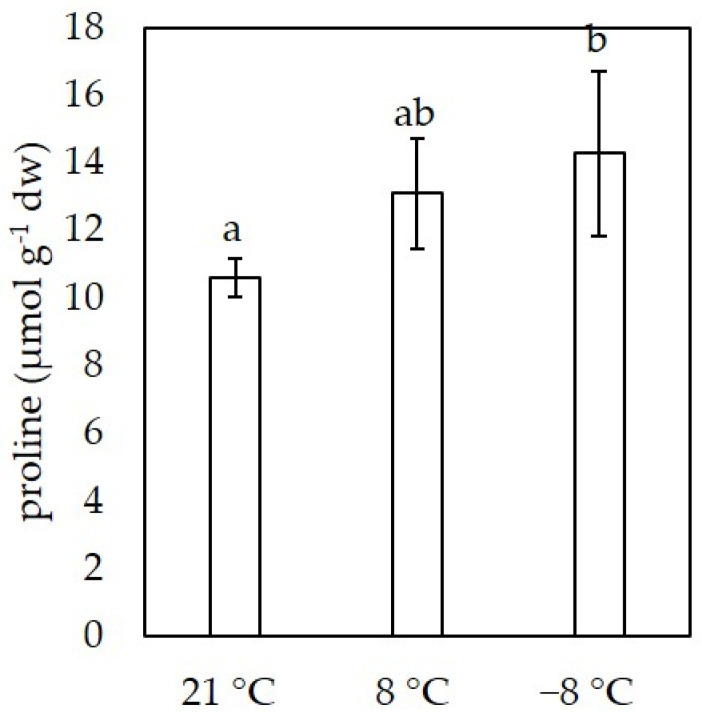
Proline content (µmol g^−1^ dw) in flat leafe kale (*B. oleracea* var. *acephala*) sprouts at different temperatures. Value marked with different letters are significantly different at *p* < 0.05.

The content of proline, an amino acid, plays an important role in plant stress response. These are shown in Figure 2. 

In control sprouts growing at 21 °C, the endogenous proline content was 10.60 ± 0.58 μmol g^−1^ dw, but the low temperature conditions triggered an accumulation of proline in sprouts. In sprouts exposed to an additional −8 °C for one hour the proline content was significantly higher (14.28 ± 2.45 μmol g^−1^ dw) than in control plants growing at 22 °C.

### 3.3. Pigments Content

Content of chlorophyll *a*, *b*, total chlorophylls, total carotenoids, as well as chlorophyll *a*/*b* and total chlorophylls/total carotenoids ratios are shown in Table 2.

As shown in Table 2, low temperature caused a significant decrease in the content of chlorophyll *a*, *b*, total chlorophylls and carotenoids. However, the ratio of chlorophyll *a*/*b* and total chlorophyll/total carotenoids remained the same and were approximately 2.5 and 4, respectively.

### 3.4. Polyphenol Content

**Figure 3:**
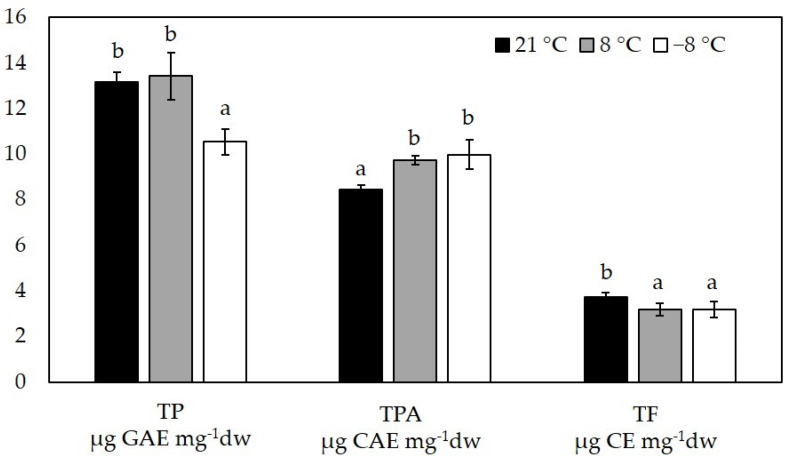
Content of total polyphenols (TP), total phenolic acids (TPA) and total flavonoids (TF) in flat leaf kale sprouts (*B. oleracea* var. *acephala*) affected by different temperatures. Value marked with different letters are significantly different at *p* < 0.05.

Content of total polyphenols, total phenolic acids and total flavonoids are shown in Figure 3.

Total phenolic content (TP) did not differ between sprouts grown at 21 °C (13.17 ± 0.41 µg GAE mg^−1^ dw) and 8 °C (13.43 ± 1.02 µg GAE mg^−1^ dw), but decreased significantly in sprouts exposed to −8 °C (10.54 ± 0.57 µg GAE mg^−1^ dw). Total flavonoids (TF) follow a similar trend and both low temperatures (8 °C and −8 °C) which caused a decrease in total flavonoid content compared to the control (3.73 ± 0.21 µg CE mg^−1^ dw). However, low temperatures caused an increase in total phenolic acids. Control plants accumulated 8.46 ± 0.18 µg CAE mg^−1^ dw, while sprouts grown at 8 °C and −8 °C had 9.74 ± 0.18 CAE mg^−1^ dw and 9.98 ± 0.65 CAE mg^−1^ dw, respectively.

### 3.5. Glucosinolates Content

**Figure 4:**
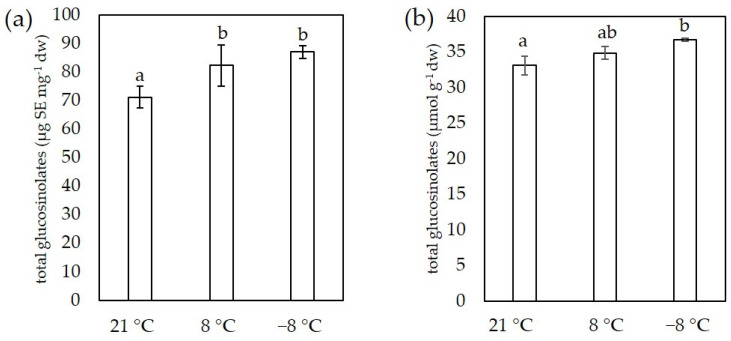
Total glucosinolates content measured spectrophotometrically (**a**) and by HPLC-DAD (**b**) in flat leaf kale sprouts (*B. oleracea* var. *acephala*) affected by different temperatures. Value marked with different letters are significantly different at *p* < 0.05.

For the determination of glucosinolates we used two methods, spectrophotometric and HPLC-DAD. The results for total glucosinolate content for both methods are shown in Figure 4.

Total glucosinolate content in sprouts grown at 21 °C was 71.18 ± 3.81 μg SE mg^−1^ dw and increased significantly to 82.32 ± 7.32 μg SE mg^−1^ dw and 87.10 ± 2.19 μg SE mg^−1^ dw in a sprouts grown at 8 °C and −8 °C, respectively. We also observed similar trends using the HPLC-DAD method. By this method, the total glucosinolate content was 33.08 ± 1.28 μmol g^−1^ dw in control samples, 34.84 μmol g^−1^ dw in sprouts grown at 8 °C, and 36.69 ± 0.21 μmol g^−1^ dw at −8 °C. This method also determines individual glucosinolates, the amounts of which are listed in Table 3.

**Figure 5:**
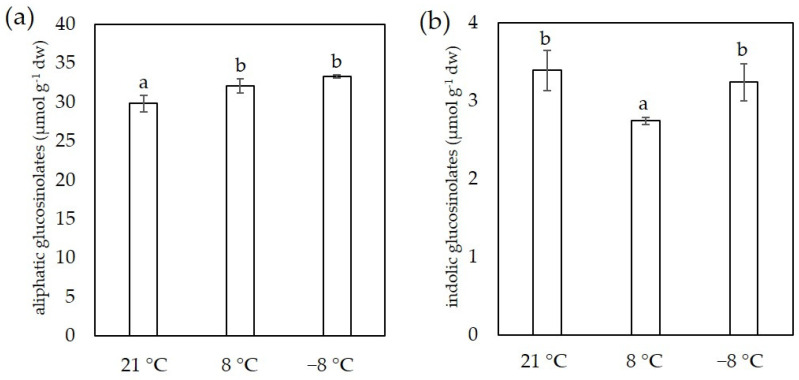
The content of aliphatic and indolic glucosinolates in flat leafe kale sprouts (*B. oleracea* var. *acephala*) affected by different temperatures measured by spectrophotometry (**a**) and HPLC-DAD (**b**) method. Value marked with different letters are significantly different at *p* < 0.05.

As shown in Table 3, the most abundant individual glucosinolate was sinigrin. The low temperature of 8 °C had no significant effect on sinigrin content, but additional exposure at −8 °C resulted in an accumulation of sinigrin. Besides sinigrin, according to our results, three other glucosinolates are present in concentrations greater than 1 μmol g^−1^: progoitrin, glucoiberin and 4-hydroxyglucobrassicin, but temperature affected their accumulation differently. The content of glucoiberin was lower at 8 °C, while that of progoitrin was higher at low temperature compared the control at 21 °C. The content of glucobrassicanapin, 4-methoxyglucobrassicin, and neoglucobrasscin content was lower at 8 °C than in sprouts grown at 21 °C or in those exposed to −8 °C. Interestingly, we detected glucoraphanin only in the sprouts grown at 8 °C. According to our results, we detected 10× more aliphatic than indolic glucosinolates (Figure 5). The content of aliphatic glucosinolates increased with decreasing temperature, while the content of indolic glucosinolates was lower in sprouts grown at 8 °C than in those grown at 21 °C.

## 4. Discussion

During growth and development, plants can be exposed to several biotic and abiotic stresses that can significantly affect yield and the content of nutrients and phytochemicals in plant foods [37]. During growth, plants are affected by several different stressors. In order to study only one specific parameter, it was necessary to develop an experimental design in which all other parameters are constant. In the present study, we germinated and grown kale sprouts in vitro (in Petri dishes) to minimize all other factors except temperature that may affect accumulation of potentially healthy phytochemicals in sprouts. The experiments were carried out in the laboratory under sterile conditions to avoid all possible contamination. Growing the sprouts in a vertical position allowed us to easily determine the lengths of roots and shoots (Table 1). As expected, low temperatures (8 °C) slowed down the growth of sprouts, which was reflected in the length of roots and yield, which were significantly lower at 8 °C. At low temperatures, roots exhibited the greatest reduction in growth, which was expected since sprouts first develop roots that later enable the plant to take up nutrients from the growing medium. Sprouts growing at 21 °C grew more uniformly compared to sprouts growing at 8 °C, as indicated by the lower standard deviation. A similar observation was reported by Pereira et al. [38] for broccoli sprouts (*B. oleracea* var. *italica*), which took almost 10 days at 11.3 °C to achieve the same growth increment obtained in <3 days at 28 °C. In this study, the authors sprouts weighing about 25 mg per sprouts, which is comparable to the weight of our sprouts.

The reduction in growth at lower temperatures indicates that the sprouts are exposed to stress and their physiological state is changing. This is also evident from the increase in proline content in sprouts growing at lower temperatures (Figure 2). Proline is a multifunctional molecule and acts as an osmoprotectant, metal chelator, antioxidant defense molecule and signaling molecule in plants [39]. Correlation between proline accumulation and low temperature stress was already reported for *Brassica* plants [40]. The physiological status of the plant can also be determined by measuring the pigment content, the level of which correlates with the photosynthetic capacity of the plants [30]. In our experiments, sprouts growing at 8 °C had significantly lower levels of chlorophyll *a*, *b*, total chlorophylls, and carotenoids than sprouts grown at 21 °C, which may indicate a reduction in photosynthetic performance. This trend has been previously reported for rice sprouts [41] and tung tree [42] and watermelon seedlings [43] exposed to low temperatures. Although the content of individual pigments was lower in our samples at lower temperatures, the ratio of chlorophyll *a*/*b* and total chlorophyll/carotenoids did not change significantly. The weight ratio of chlorophyll *a*/*b* is an indicator of functional pigmentation and the equipment and light adaptation of the photosynthetic apparatus, while the ratio of total chlorophyll/total carotenoids is an indicator of the green coloration of plants [30].

Carotenoids are a group of pigments that are common in photosynthetic organisms and include more than 850 different structures [44]. They are present in the photosynthetic organs of plants and also in the green parts of sprouts. In green vegetables, their presence is often masked by the green color of chlorophylls, but green vegetables are considered a good source of carotenoids [45]. After digestion in humans, they can have numerous health benefits. They are precursors of vitamin A, photoprocessors, antioxidants, immunity enhancers, and contribute to reproduction [44]. In this experiment, the total carotenoid content (Table 2) is comparable to values previously reported for kale sprouts [2], but the content in the sprouts is lower than the content of carotenoids in more mature kale plants [46,47] from the same variety. These results are in line with the report of Wojdyło et al. [21] who compare carotenoid content in sprouts and microgreen and found several times more carotenoids in microgreens. This is not surprising because older plants have a fully developed photosynthetic apparatus and accumulate more pigments. In our experiment in sprouts grown under low temperature we determinate less carotenoids than in sprouts grown at 21 °C. It was already reported that low temperatures affect the genes related with carotenoids synthesis but likely the dynamic of carotenoid changes may be related with cultivar or plants development stage [48]. For, example, unlike our results Jurkow et al. [47] reported an increase in carotenoid content in fully mature kale plants after moderate (−5 °C) and severe frost (−15 °C). This may indicate that mature plants are less sensitive to low temperatures than seedlings.

Polyphenols are a well-known group of phytochemicals associated with numerous health benefits and contribute significantly to the biological activity of *Brassica* plants in synergy with other compounds [8]. They also play an important role in plant–environment interaction and their content can be significantly affected by external factors such as low temperatures [49]. In our experiment, we determined the total amount of polyphenols, phenolic acids and flavonoids in kale sprouts. The total content of polyphenols and flavonoids was comparable to the content previously reported for kale sprouts [2,50], while in this experiment we found a 2× higher total content of phenolic acids than we had previously reported for kale sprouts [2]. The total phenolic content of sprouts grown at 21 °C and 8 °C did not differ significantly, while additional exposure to −8 °C resulted in a significant decrease in total phenolic content. In our experiments, total phenolic acid content increased at low temperatures, while total flavonoid content decreased. Swieca and Baraniak [51] reported that exposure of lentil sprouts to 4 °C for 1 h increased the total phenolic and flavonoid content and that low temperature can be an effective trigger for phytochemicals accumulation. In a report by Ragusa et al. [52], a direct comparison of total polyphenolic compounds in broccoli and arugula sprouts grown at different temperatures (10 °C, 20 °C, and 30 °C) revealed a different trend; in broccoli, total polyphenolic compounds increased with higher temperature, while in arugula, they were highest at 10 °C and decreased with higher temperatures. The changes in polyphenolic compounds at low temperatures probably depend on plant species/cultivars, their cold tolerance and growth stage [52]. 

The third group of phytochemicals which we determine are glucosinolates, sulfur- and nitrogen-containing specialized metabolites in plants, which are common in the Brassicaceae and related plant families [53]. Glucosinolates are not biologically active, but are hydrolyzed by myrosinases to indoles, thiocyanates, and isothiocyanates, which have numerous biological potentials [54]. In addition, glucosinolates may contribute to the flavor of vegetables and sprouts [55]. The total content of glucosinolates determined by spectrophotometric method was several times higher than in mature plants that we measured in previous studies using the same method [46,47]. This is consistent with the study of Drozdowska et al. [13] on red cabbage, where they reported that young shoots are a better source of glucosinolates than mature plants at the stage of intensive growth. In addition, Pereira et al. [38] reported that glucosinolates content in broccoli sprouts decline as sprouts germinate and develop, with glucosinolates content consistently higher at the younger stages of development. Results on the presence of individual glucosinolates showed that there were 10× more aliphatic glucosinolates than indolic glucosinolates in kale sprouts, which is consistent with our previous results for kale sprouts [2]. The predominant glucosinolate was sinigrin, which has already been reported as the most abundant glucosinolate in kale sprouts [2,56,57] and more mature kale plants [46,58], suggesting that the ratio of the different individual glucosinolates is genetically determined. Results from both methods for total glucosinolate content showed that glucosinolates increased at lower temperatures, which is consistent with our previous results for 9-week-old kale plants exposed to chilling and freezing temperatures [46]. The increase in total glucosinolates content in sprouts is probably related to the increase in aliphatic glucosinolates. However, the content of individual glucosinolates developed differently in kale sprouts at low temperatures, probably due to their different involvement in low temperature stress.

## 5. Conclusions

Kale sprouts (*B. oleracea* var. *acephala*) can be a convenient and easily utilized source of phytochemicals from the group of carotenoids, polyphenols, and glucosinolates. However, growing conditions may affect this content. In the present study, we investigated the effect of low temperatures on the physiological status and phytochemical content of kale sprouts (*B. oleracea* var. *acephala*) and conducted five independent laboratory experiments. Low temperatures cause stress to the sprouts, as evidenced by increased proline content and lower yield. Growing the sprouts at 8 °C and freezing them at −8 °C for one hour cause a significant decrease in the content of carotenoids and total flavonoids, while the total content of phenolic acids and glucosinolates increases. All in all, our results show that low temperatures significantly affect the content of phytochemicals in flat leaf kale sprouts (*B. oleracea* var. *acephala*).

## Figures and Tables

**Table 1:** Mass per sprout, root and shoots lenght of flat leafe kale (*B. oleracea* var. *acephala*) sprouts grown at 21 °C (control) and 8 °C. Value marked with asterisk (*) are significantly different from the control at *p* < 0.05.

	21 °C	8 °C
	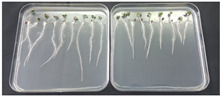	
Mass 1 seedling (mg)	26.83 ± 3.90	23.40 ± 5.31 *
Root length (mm)	54.95 ± 10.75	35.62 ± 8.82 *
Shoots length (mm)	6.95 ± 1.91	6.19 ± 2.03

**Table 2:** Content of pigments in flat leafe kale (*B. oleracea* var. *acephala*) sprouts affected by different temperatures. Value marked with different letters are significantly different at *p* < 0.05.

	21 °C	8 °C	−8 °C
Chlorophyll *a* (µg mg−^1^ dw)	1.98 ± 0.32 ^b^	1.43 ± 0.15 ^a^	1.47 ± 0.35 ^a^
Chlorophyll *b* (µg mg^−1^ dw)	0.81 ± 0.09 ^b^	0.58 ± 0.06 ^a^	0.59 ± 0.12 ^a^
Total chlorophylls (µg mg^−1^ dw)	2.79 ± 0.38 ^b^	2.02 ± 0.20 ^a^	2.06 ± 0.47 ^a^
Total carotenoids (µg mg^−1^ dw)	0.64 ± 0.06 ^b^	0.51 ± 0.04 ^a^	0.51 ± 0.06 ^a^
Chlorophyll *a*/Chlorophyll *b*	2.46 ± 0.28 ^a^	2.45 ± 0.17 ^a^	2.50 ± 0.15 ^a^
Chlorophylls/carotenoids	4.34 ± 0.33 ^a^	3.95 ± 0.16 ^a^	3.97 ± 0.50 ^a^

**Table 3:** Concentration (in μmol g^−1^ dw) of individual glucosinolates in flat leafe kale sprouts (*B. oleracea* var. *acephala*) affected by different temperatures. Value marked with different letters are significantly different at *p* < 0.05.

μmol g^−1^ dw	21 °C	8 °C	−8 °C
Glucoiberin	3.94 ± 0.17 ^b^	3.13 ± 0.09 ^a^	3.78 ± 0.04 ^b^
Progoitrin	4.69 ± 0.10 ^a^	5.85 ± 0.20 ^b^	4.92 ± 0.35 ^a^
Sinigrin	17.95 ± 0.80 ^a^	16.82 ± 0.96 ^a^	20.55 ± 0.49 ^b^
Glucoraphanin	nd	2.76 ± 0.15	nd
Gluconapin	0.69 ± 0.07 ^a^	0.66 ± 0.04 ^a^	0.86 ± 0.01 ^b^
4-hydroxyglucobrassicin	1.73 ± 0.13 ^a^	1.65 ± 0.03 ^a^	1.98 ± 0.19 ^a^
Glucobrassicanapin	0.66 ± 0.05 ^b^	0.50 ± 0.02 ^a^	0.67 ± 0.05 ^b^
4-methoxyglucobrassicin	0.14 ± 0.01 ^b^	0.09 ± 0.01 ^a^	0.13 ± 0.02 ^b^
Neoglucobrasscin	0.71 ± 0.05 ^b^	0.51 ± 0.06 ^a^	0.61 ± 0.06 ^ab^

nd = not detected.

## Data Availability

All additional data are available upon request.

## Supplementary Materials

The following supporting information can be downloaded at: https://www.mdpi.com/article/10.3390/foods11030264/s1, Figure S1: Representative chromatogram of glucosinolates analysis.
