# Supplementary figures and images for: Low Temperatures Affect the Physiological Status and Phytochemical Content of Flat Leaf Kale (Brassica oleracea var. acephala) Sprouts
Source: Foods. 2022 Jan 19;11(3):264. doi: 10.3390/foods11030264 (PMC8834612; doi:10.3390/foods11030264)

Figure S1. Representative chromatogram of glucosinolates analysis

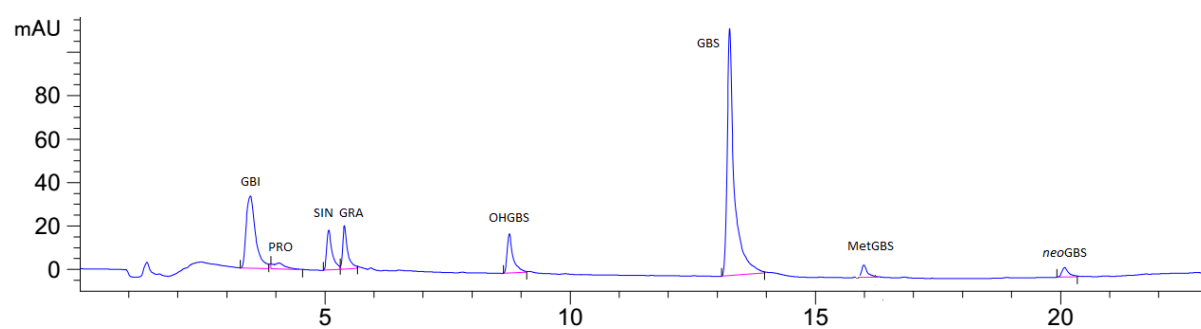

Supplement: Supplementary file 1 [file foods-11-00264-s001.zip › foods-1551210-supplementary.pdf]
